# The Effect of Aging on Brain Glucose Metabolic Connectivity Revealed by [^18^F]FDG PET-MR and Individual Brain Networks

**DOI:** 10.3389/fnagi.2021.798410

**Published:** 2022-02-09

**Authors:** Nathalie Mertens, Stefan Sunaert, Koen Van Laere, Michel Koole

**Affiliations:** ^1^Nuclear Medicine and Molecular Imaging, Department of Imaging and Pathology, KU Leuven, Leuven, Belgium; ^2^Translational MRI, Department of Imaging and Pathology, KU Leuven, Leuven, Belgium; ^3^Department of Radiology, University Hospitals Leuven, Leuven, Belgium; ^4^Division of Nuclear Medicine, University Hospitals Leuven, Leuven, Belgium

**Keywords:** metabolic connectivity, healthy aging, individual brain network, functional connectivity, [^18^F]FDG PET

## Abstract

Contrary to group-based brain connectivity analyses, the aim of this study was to construct individual brain metabolic networks to determine age-related effects on brain metabolic connectivity. Static 40–60 min [^18^F]FDG positron emission tomography (PET) images of 67 healthy subjects between 20 and 82 years were acquired with an integrated PET-MR system. Network nodes were defined by brain parcellation using the Schaefer atlas, while connectivity strength between two nodes was determined by comparing the distribution of PET uptake values within each node using a Kullback–Leibler divergence similarity estimation (KLSE). After constructing individual brain networks, a linear and quadratic regression analysis of metabolic connectivity strengths within- and between-networks was performed to model age-dependency. In addition, the age dependency of metrics for network integration (characteristic path length), segregation (clustering coefficient and local efficiency), and centrality (number of hubs) was assessed within the whole brain and within predefined functional subnetworks. Overall, a decrease of metabolic connectivity strength with healthy aging was found within the whole-brain network and several subnetworks except within the somatomotor, limbic, and visual network. The same decrease of metabolic connectivity was found between several networks across the whole-brain network and the functional subnetworks. In terms of network topology, a less integrated and less segregated network was observed with aging, while the distribution and the number of hubs did not change with aging, suggesting that brain metabolic networks are not reorganized during the adult lifespan. In conclusion, using an individual brain metabolic network approach, a decrease in metabolic connectivity strength was observed with healthy aging, both within the whole brain and within several predefined networks. These findings can be used in a diagnostic setting to differentiate between age-related changes in brain metabolic connectivity strength and changes caused by early development of neurodegeneration.

## Introduction

[^18^F]FDG positron emission tomography (PET) is a valuable molecular neuroimaging technique to study the glucose metabolism in the human brain which in turn serves as a proxy for neuronal activity. Many studies have shown a progressive decrease of cerebral [^18^F]FDG uptake with aging, mainly observed in the medial frontal lobe and anterior cingulate cortex (Fujimoto et al., 2008; Knopman et al., 2014; Yoshizawa et al., 2014; Kakimoto et al., 2016; Ishibashi et al., 2017; Malpetti et al., 2017; Van Aalst et al., 2021). Whether these age-related changes are a linear or quadratic function of adult age with the latter showing accelerated changes in the elderly is still under debate. In parallel, structural and functional cerebral changes have been detected during the lifespan by different groups using MRI techniques. Overall, these studies showed increased gray matter (GM) atrophy observed by voxel-based morphometry MRI analyses (Good et al., 2001; Allen et al., 2005; Smith et al., 2007; Bagarinao et al., 2018) and reduced cerebral structural integrity assessed by diffusion tensor imaging (DTI) (Moseley, 2002; Head et al., 2004; Sullivan and Pfefferbaum, 2006) and differences in brain activation patterns using functional MRI (fMRI) (Grady, 2012; Avelar-Pereira et al., 2017) due to aging. To study these aging effects on brain structure and function, brain connectivity analysis has proven to be a very useful approach as it reveals important information about connections and interactions between different brain regions and allows to study the brain from a topological viewpoint. To assess brain connectivity, graph theoretical methods are generally applied which model the brain using a weighted, undirected graph. This way, a wide range of graph-based connectivity measures, reflecting both local and global brain connectivity, is extracted to quantify the underlying network topology. In literature, the majority of connectivity findings are derived from DTI and fMRI studies, which provide information on axonal pathways, or on correlations between the blood-oxygen-level-dependent (BOLD)-signal time course of different brain regions (Betzel et al., 2014; Damoiseaux, 2017; Frau-Pascual et al., 2021). In contrast to structural and functional connectivity, brain metabolic connectivity findings using [^18^F]FDG PET are mainly based on group-level analyses (Sala and Perani, 2019) where correlations between regional uptake values across subjects are used as connectivity measures between different brain regions. However, a novel approach using the Kullback–Leibler divergence similarity estimation (KLSE) was recently introduced to generate an individual brain metabolic network for a single subject using static [^18^F]FDG PET imaging (Wang et al., 2020a). This technique assumed that brain regions with similar glucose metabolism are highly interconnected while brain regions with differences in glucose metabolism have a lower connectivity strength. To determine the connectivity strength between two regions, KLSE was used to compare the intra-regional distribution of PET uptake values between different regions. Using these metabolic connectivity strengths, the approach successfully predicted the individual risk of progression from mild cognitive impairment (MCI) to Alzheimer’s disease (AD) (Wang et al., 2020a). The aim of this study was to apply this novel technique on [^18^F]FDG PET-MR data of a cohort of 67 healthy controls, covering an age range of 20–82 years, to evaluate age-related effects on graph-based connectivity measures for network integration, segregation, and centrality. We evaluated these age effects on the level of both the whole brain and different functional brain networks where we considered networks which represent the intrinsic functional connectivity of the cerebral cortex. This study is also the first step toward using these metrics in a diagnostic setting where it is mandatory to discriminate effects of healthy aging from early development of neurodegeneration as aging is the primary risk factor for many neurodegenerative disorders (Hou et al., 2019).

## Materials and Methods

### [^18^F]FDG PET-MR Imaging

A total of 67 healthy volunteers (33 males and 34 females; age: 52 ± 17 years, range 20–82 years) were recruited prospectively between December 2015 and February 2017. The main exclusion criteria for this study were major internal pathology or having (had) cancer, having a first-degree relative with dementia, a history of important neurological and/or psychiatric disorders, and substance abuse or current use of centrally acting medication. Subjects underwent a neurological examination resulting in a Mini-Mental State Examination (MMSE) score ≥ 28 and a score of ≤9 on the Beck’s Depression Inventory (BDI) for all subjects. This study was approved by the local ethics committee of UZ Leuven Gasthuisberg, and all participants gave written informed consent.

Subjects received an intravenous bolus injection of [^18^F]FDG (152 ± 10 MBq) and underwent a simultaneous [^18^F]FDG PET-MR scan (General Electric Healthcare Signa PET-MR). Listmode data acquired between 40 and 60 min were rebinned in four frames of 5 min and corrected for motion. Sinograms were corrected for dead time, random, and scatter, while a proprietary template-based MR-based attenuation correction (MRAC) was used for attenuation correction. Each frame was reconstructed using ordered subset expectation maximization (OSEM, 28 subsets and 4 iterations) and included time of flight (TOF) information, resolution modeling, and a Gaussian post-smoothing with a full width half maximum (FWHM) of 4.5 mm. The multi-frame PET data were rescaled to standardized uptake values (SUV) and averaged to obtain a static SUV PET image.

Simultaneous with the PET acquisition, a 3D volumetric 3 Tesla T1 weighted BRAVO MR sequence was acquired using an 8-channel phased-array coil (plane: sagittal; echo time (TE): 3.2 ms; repetition time (TR): 8.5 ms; inversion time (TI): 450 ms; flip angle: 12°; receiver bandwidth: 31.2 kHz; NEX: 1; voxelsize: 1 mm × 1 mm × 1 mm) followed by a 3D T2 weighted FLAIR sequence (plane: sagittal; TE: 130 ms; TR: 8,500 ms; TI: 2,298 ms; voxelsize: 1 mm × 1 mm × 1.4 mm).

### Individual Brain Metabolic Connectivity Networks

[^18^F]FDG PET images were spatially normalized using a non-linear normalization using the CAT12 toolbox of Statistical Parametric Mapping (SPM12; Welcome Trust Centre for Neuroimaging, University College, London, United Kingdom) and smoothed with a Gaussian filter of 8 mm. For each subject, [^18^F]FDG uptake was normalized to the total uptake in the GM. Subject specific tissue probability maps for GM, white matter (WM), and cerebrospinal fluid (CSF) were derived based on the 3D T1-weighted MR data in SPM12 and used to delineate volume of interest (VOI) defined by the Schaefer atlas (Schaefer et al., 2018). This functional atlas includes the frontoparietal (4 VOIs) network together with seven functional subnetworks containing the visual (13 VOIs), somatomotor (14 VOIs), dorsal attention (13 VOIs), salience and ventral attention (14 VOIs), limbic (5 VOIs), control (16 VOIs), and default mode (21 VOIs) network (Supplementary Table 1).

For depicting an individual metabolic network for each subject, the 100 brain parcels, determined by the Schaefer atlas, were considered as nodes, and the [^18^F]FDG uptake in each pair of nodes was used to generate a metabolic correlation matrix. This was performed by extracting the intensity values of voxels within each node to estimate the probability density function (PDF) of intensity values for that parcel. All PDFs were estimated using brain parcels in MNI space containing minimum 800 voxels. Furthermore, the PDF for each parcel was estimated using the kernel density estimation (KDE) with optimal bandwidths for the number of voxels of that specific parcel chosen automatically using the diffusion Botev method as implemented in the KDE-diffusion toolbox in Python version 3.9 (Botev et al., 2010). In addition, PDFs were estimated in a standardized histogram space with a fixed range of values and a fixed bin size for all parcels and all subjects. Then, the KLSE method was used to estimate the similarity between the PDFs of two nodes and construct a correlation matrix which represents the pairwise metabolic connections or edges. In general, KLSE is based on the KL divergence (*D*_KL_) between two PDFs. However, to have a symmetric measure, the following variation of *D*_KL_ was used:


$$D_{K\,L}\,\left(P,Q\right)=\int_{x}\left(P\,\left(x\right)\,l\,o\,g\,\frac{P\,\left(x\right)}{Q\,\left(x\right)}+Q\,\left(x\right)\,l\,o\,g\,\frac{Q\,\left(x\right)}{P\,\left(x\right)}\right)\,dx$$

where *P* and *Q* are two PDFs defined on the same *x* range. Finally, the metabolic connectivity strength between two nodes was calculated as the KL similarity (KLS) measure as follows:


$$K\,L\,S\,\left(P,Q\right)=e^{-D_{K\,L}\,\left(P,Q\right)}.$$

This way, an undirected weighted metabolic connectivity matrix was estimated for each subject and quantified using graph-based connectivity metrics without applying a threshold to generate a binarized connectivity matrix.

### Brain Metabolic Connectivity Metrics

Several graph theory metrics of metabolic connectivity were calculated to characterize global and nodal connectivity. Global connectivity of each network was assessed using the mean connectivity strength and the characteristic path length, while nodal connectivity was assessed using the clustering coefficient and the local efficiency. All nodal metrics were averaged over all pairs of nodes in order to examine network characterization of the whole network. Furthermore, to assess network centrality, four nodal metrics were used, being the degree, characteristic path length, clustering coefficient, and betweenness centrality to determine central nodes within the network, called hubs. To calculate the clustering coefficient and the local efficiency, a generalization for weighted undirected graphs was used as proposed by Wang et al. (2017), while all other metrics were calculated using the brain-connectivity toolbox in Python (Rubinov and Sporns, 2010).

First, to quantify the connectivity within each individual network, the average metabolic connectivity strength over all pairs of nodes of each network was determined for the whole-brain network. In addition to this whole-brain connectivity measure, the metabolic connectivity strength within each functional subnetwork was assessed as well as between-network metabolic connectivity strengths. The average within-network connectivity strength was calculated by averaging the connectivity values over all nodes within each functional subnetwork, while between-network connectivity strengths were generated by averaging the connectivity values over all nodes within two functional subnetworks (Varangis et al., 2019).

Then, two types of connectivity metrics were calculated for each metabolic network. First, the characteristic path length of each network was calculated as the measure of functional integration of the brain network. Second, the average clustering coefficient over all nodes, reflecting the average prevalence of clustered connectivity around individual nodes, as well as the average local efficiency which represents the average strength of local connectedness within neighboring nodes, were calculated as measures of functional segregation of the brain network. Measures of network integration and segregation were calculated for the whole-brain network, as well as within each functional subnetwork.

Finally, hubs within each individual network were identified based on the hub score using four criteria which are determined based on whether the node belongs to the top 20% of nodes (a) showing the highest degree, (b) showing the lowest path length, (c) showing the lowest clustering coefficient, and (d) showing the highest betweenness centrality. If the hub score was at least 2, the node was considered a hub (Ran et al., 2020). As such, the number of hubs is a measure of functional centrality in the corresponding network. To examine a potential network reorganization during aging, we divided our study population in a group of young (*n* = 22, age: 32 ± 7 years), middle-aged (*n* = 22, age: 51 ± 5 years), and elderly (*n* = 23, age: 69 ± 6 years) healthy volunteers and compared the number of hubs within the whole brain and within functional subnetworks between these groups.

### Statistics

A multiple linear regression model was used to assess the effect of age on the different network connectivity metrics within the whole-brain network, as well as within and between different functional subnetworks. Both a linear and second-order polynomial (quadratic) age dependency of connectivity metrics were considered while the sum-of-squares *F* test was used to select the most appropriate model. Goodness of fit was reported using the coefficient of multiple correlation *r*. All statistical analyses were performed with Prism (version 9, GraphPad, San Diego, CA, United States) using a significance level of *p* < 0.05. Then, significant multiple linear regression models were used to assess% differences in connectivity metrics during the adult lifespan by comparing a 20-year-old subject with an 80-year-old subject.

## Results

An overview of linear and quadratic age effects on metabolic connectivity metrics within the whole-brain network and the functional subnetworks are given in Table 1. In addition, representative mean and coefficient of covariation of metabolic connectivity matrices for three age groups (young, middle-aged and old, respectively) are shown in Supplementary Figure 1.

**TABLE 1:** Overview of linear and quadratic age effects (age and age^2^, respectively) on metabolic network characteristics within the whole-brain network, as well as within functional subnetworks obtained from a multiple linear regression model.

	Mean connectivity strength	Characteristic path length	Average clustering coefficient	Average local efficiency
Whole brain network	Age	Age	Age	Age
Frontoparietal network	Age^2^	Age^2^	Age^2^	Age^2^
Default mode network	Age	Age	Age	Age^2^
Control network	Age	Age	Age	Age
Dorsal attention network	Age	Age	Age	Age
Ventral attention network	Age	Age	Age	Age
Somatomotor network	/	/	/	/
Limbic network	/	/	/	/
Visual network	/	/	/	/

### Age Effects on Mean Metabolic Connectivity Strength

**FIGURE 1:**
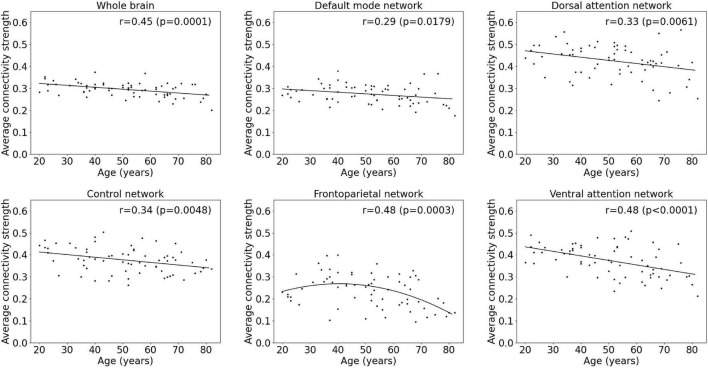
Multiple linear regression model of average metabolic connectivity strength with age within the whole-brain network and within functional subnetworks.

**FIGURE 2:**
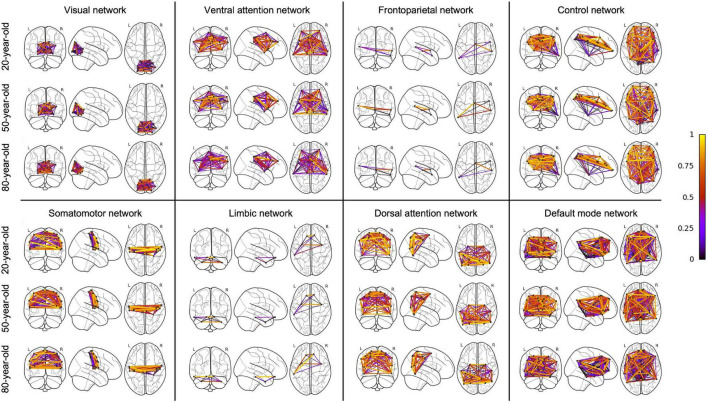
Connectome for a young and elderly healthy subject within the ventral attention network and the somatomotor network with an upper threshold of 0.80 for the metabolic connectivity strength, showing a decreased metabolic connectivity strength with age in the ventral attention network but not in the somatomotor network.

An overview of regression analysis results assessing the average metabolic connectivity strength as function of age is given in Table 2 and Figure 1 for the whole-brain network as well as for different functional subnetworks. Within the whole network, a linear decreasing age effect on the metabolic connectivity strength was found (*p* = 0.0001, *r* = 0.45), resulting in a decrease of 16.3% during the adult lifespan. This decrease in metabolic strength within the brain network was also observed when comparing the distribution of metabolic strength of a 20-year-old with an 80-year-old subject (Supplementary Figure 2). For the predefined functional subnetworks, a quadratic decreasing age effect on the metabolic connectivity strength was found within the frontoparietal network (*p* = 0.0008, *r* = 0.46), showing a decrease in metabolic strength of 41.2% between a 20-year-old and 80-year-old subject. In the default mode, control, dorsal attention, and ventral attention network, a linear decrease of metabolic connectivity strength with age was found (*p* = 0.0179, *r* = 0.29; *p* = 0.0048, *r* = 0.34; *p* = 0.0061, *r* = 0.33; and *p* < 0.0001, *r* = 0.48, respectively), resulting in a decrease of 14.9, 17.2, 18.4, and 28.3%, respectively, during the adult lifespan. In contrast, no effect of age was found in the somatomotor, limbic, and visual network. Representative connectome networks of the ventral attention and the somatomotor network with an upper connectivity threshold of 0.80 of a young and an old healthy subject are given in Figure 2, showing lower connectivity within the older subject compared with the younger subject in the ventral attention, dorsal attention, frontoparietal, control, and default mode network, but not in the somatomotor, limbic, and visual network.

**TABLE 2:** Overview of multiple linear regression analyses to model network metrics as function of age within the whole-brain network and within functional subnetworks.

	*p*-Value	*r*-Value	ß0	ß1	ß2	20y	80y	%Diff
**Mean connectivity strength**								
Whole brain network	**0.0001**	0.45	**0.34**	**-0.88E-03**	/	0.32	0.27	–16.3
Frontoparietal network	**0.0003**	0.48	0.13	**6.80E-03**	**–0.08E-03**	0.23	0.14	–41.2
Default mode network	**0.0179**	0.29	**0.31**	**-0.739E-03**	/	0.30	0.25	–14.9
Control network	**0.0048**	0.34	**0.44**	**–1.182E-03**	/	0.41	0.34	–17.2
Dorsal attention network	**0.0061**	0.33	**0.50**	**–1.442E-03**	/	0.47	0.38	–18.4
Ventral attention network	**<0.0001**	0.48	**0.48**	**–2.06E-03**	/	0.44	0.31	–28.3
Somatomotor network	0.9922	/	/	/	/	/	/	/
Limbic network	0.4787	/	/	/	/	/	/	/
Visual network	0.2913	/	/	/	/	/	/	/
**Characteristic path length**								
Whole brain network	**<0.0001**	0.49	**2.29**	**5.26E-03**	/	2.39	2.71	13.2
Frontoparietal network	**0.0001**	0.49	**4.56**	**–94.12E-03**	**1.21E-03**	3.16	4.78	51.4
Default mode network	**0.0036**	0.35	**2.43**	**7.19E-03**	/	2.57	3.01	16.8
Control network	**0.0121**	0.30	**1.97**	**4.78E-03**	/	2.07	2.35	13.9
Dorsal attention network	**0.0031**	0.36	**1.69**	**6.28E-03**	/	1.81	2.19	20.8
Ventral attention network	**<0.0001**	0.51	**1.72**	**11.11E-03**	/	1.94	2.61	34.4
Somatomotor network	0.9621	/	/	/	/	/	/	/
Limbic network	0.5648	/	/	/	/	/	/	/
Visual network	0.4884	/	/	/	/	/	/	/
**Average clustering coefficient**								
Whole brain network	**0.0001**	0.45	**0.36**	**–0.92E-03**	/	0.34	0.28	–16.4
Frontoparietal network	**0.0003**	0.47	**0.23**	**9.40E-03**	**–0.11E-03**	0.37	0.28	–24.8
Default mode network	**0.0314**	0.26	**0.34**	**–0.74E-03**	/	0.33	0.29	–13.5
Control network	**0.0048**	0.34	**0.49**	**–1.31E-03**	/	0.46	0.39	–16.9
Dorsal attention network	**0.0117**	0.31	**0.58**	**–1.54E-03**	/	0.55	0.45	–16.9
Ventral attention network	**<0.0001**	0.47	**0.55**	**–2.29E-03**	/	0.51	0.37	–27.0
Somatomotor network	0.9839	/	/	/	/	/	/	/
Limbic network	0.9913	/	/	/	/	/	/	/
Visual network	0.2458	/	/	/	/	/	/	/
**Average local efficiency**								
Whole brain network	**<0.0001**	0.50	**0.24**	**–0.60E-03**	/	0.23	0.20	–15.5
Frontoparietal network	**0.0011**	0.44	0.05	**8.31E-03**	**–0.10E-03**	0.18	0.08	–53.4
Default mode network	**0.0134**	0.36	**0.15**	2.22E-03	**–0.03E-03**	0.18	0.15	–16.1
Control network	**0.0026**	0.36	**0.29**	**–0.93E-03**	/	0.27	0.22	–20.4
Dorsal attention network	**0.0096**	0.31	**0.34**	**–1.04E-03**	/	0.32	0.26	–19.5
Ventral attention network	**0.0001**	0.46	**0.29**	**–1.36E-03**	/	0.27	0.19	–30.5
Somatomotor network	0.9425	/	/	/	/	/	/	/
Limbic network	0.1944	/	/	/	/	/	/	/
Visual network	0.8200	/	/	/	/	/	/	/

*Multiple linear regressions are described as Y = ß0 + ß1.age + ß2.age^2^. Regression p-values, overall r-values, and regression coefficients are given. Significant p-values and coefficients are given in bold. Metric values and differences between an 80-year-old and 20-year-old subject are also given.*

Finally, 26 out of 36 (72%) between-network metabolic connectivity strengths showed a significant decrease with age (Supplementary Table 2). In 7 out of 36 (19%) between-network connectivity strengths, the quadratic model was the preferred model to model age effects, while 19 out of 36 (53%) between-networks showed a linear decrease with age.

### Age Effects on Functional Integration Metrics

**FIGURE 3:**
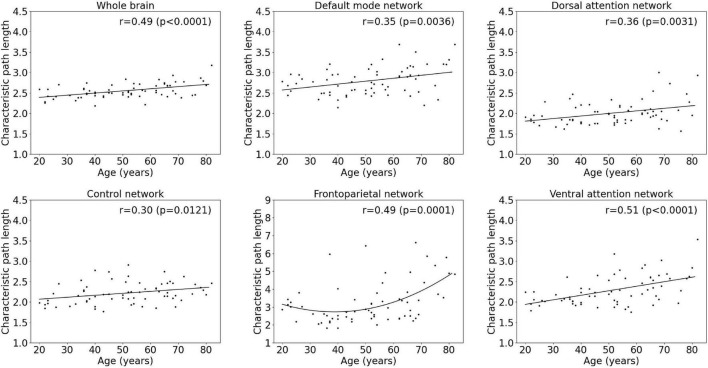
Multiple linear regression model of the characteristic path length with age within the whole-brain network and within functional subnetworks.

Results of the regression analyses to model a functional integration metric assessed by the characteristic path length as a function of age within the whole-brain network and within functional subnetworks are given in Table 2 and Figure 3. For the characteristic path length, a linear increasing effect of age was found within the whole-brain network (*p* < 0.001, *r* = 0.67), resulting in an increase of 13.2% during the adult lifespan. Within functional subnetworks, a quadratic increasing effect of age was found within the frontoparietal network (*p* = 0.0001, *r* = 0.49). During the adult lifespan, an increase in a characteristic path length of 51.4% was found within this network. Otherwise, in the default mode, control, dorsal attention, and ventral attention network, a linear increase of network integration was found with age as assessed by the characteristic path length (*p* = 0.0036, *r* = 0.35; *p* = 0.0121, *r* = 0.30; *p* = 0.0031, *r* = 0.36; and *p* < 0.0001, *r* = 0.51, respectively). Comparing the characteristic path length of a 20-year-old and 80-year-old subject, an increase of 16.8, 13.9, 20.8, and 34.4% was found in the default mode, control, dorsal attention, and ventral attention network, respectively. In contrast, no effect of age on the characteristic path length was found in the somatomotor, limbic, and visual network.

### Age Effects on Functional Segregation Metrics

**FIGURE 4:**
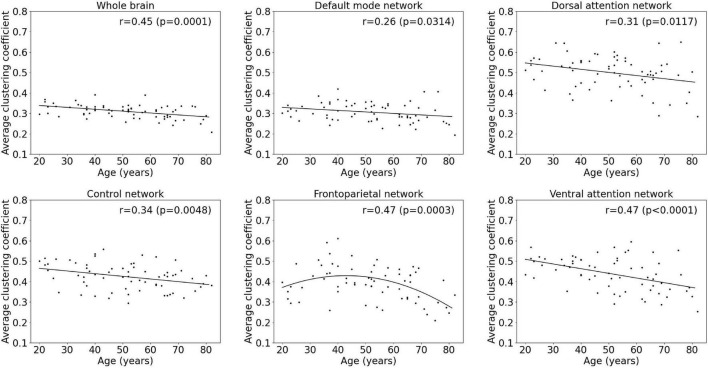
Multiple linear regression model of the average clustering coefficient with age within the whole-brain network and within functional subnetworks.

**FIGURE 5:**
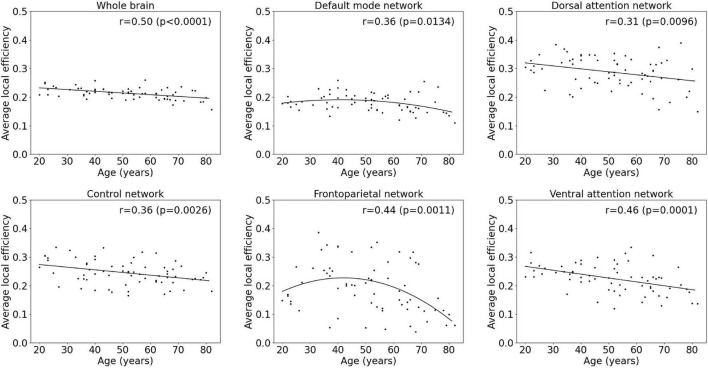
Multiple linear regression model of the average local efficiency with age within the whole-brain network and within functional subnetworks.

Results of the regression analyses to model functional segregation metrics assessed by the average clustering coefficient and the average local efficiency, as function of age within the whole-brain network and within functional subnetworks, are given in Table 2 and Figures 4, 5. For both the average clustering coefficient and the average local efficiency, a linear decreasing effect of age was found within the whole-brain network (*p* = 0.0001, *r* = 0.45 and *p* < 0.0001, *r* = 0.50). This resulted in a decrease of 16.4 and 15.5%, respectively, in terms of average clustering coefficient and average local efficiency during the adult lifespan. For the functional subnetworks, a decreasing quadratic effect with age was observed in terms of average local efficiency within the frontoparietal network (*p* = 0.0011, *r* = 0.44), as well as within the default mode network (*p* = 0.0134, *r* = 0.36). Within these networks, a decrease in the average local efficiency of 53.4 and 16.1% was observed between a 20-year-old and 80-year-old subject. For the average clustering coefficient, a decreasing quadratic effect with age was also found within the frontoparietal network (*p* = 0.0003, *r* = 0.47), while a decreasing linear effect with age was observed within the default network (*p* = 0.0314, *r* = 0.26). During the adult lifespan, a decrease in the average clustering coefficient of 24.8 and 13.5%, respectively, was observed within these networks. Furthermore, a decreasing linear age effect of network segregation was also found in the control (*p* = 0.0048, *r* = 0.34), dorsal attention (*p* = 0.0117, *r* = 0.31), and ventral attention network (*p* < 0.0001, *r* = 0.47) as assessed by the average clustering coefficient. For these subnetworks, the average local efficiency also showed a decreasing linear effect with age (*p* = 0.0026, *r* = 0.36; *p* = 0.0096, *r* = 0.31; and *p* = 0.0001, *r* = 0.46). Comparing a 20-year-old subject with an 80-year-old subject showed a decrease in the average clustering coefficient of 16.9%, 16.9%, and 27.0% respectively, and a decrease in the average local efficiency of 20.4, 19.5, and 30.5%, respectively. Again, no effect of age was found in the somatomotor, limbic, and visual network.

### Age Effects on Functional Centrality Metrics

The median and interquartile range (IQR) of the absolute number of hubs within the whole brain as well as within the functional subnetworks is given in Table 3. Within the whole brain, a linear regression analysis did not show an effect of age on the number of hubs (*p* = 0.20). In addition, no age-related reorganization was observed as the number of hubs within the whole brain and functional subnetworks remained stable in a young, middle-aged, and old group (Table 3).

**TABLE 3:** Overview of number of hubs within the whole-brain network, as well as within functional subnetworks.

	All subjects	Young	Middle-aged	Old
Whole brain network	19 (18–21)	20 (18–21)	19 (18–21)	20 (19–22)
Default mode network	4 (3–5)	4 (3–4)	4 (3–5)	4 (3–5)
Control network	4 (3–5)	4 (3–4)	3 (2–4)	4 (3–5)
Dorsal attention network	3 (2–4)	4 (3–5)	3 (2–4)	3 (3–4)
Somatomotor network	3 (2–4)	2 (1–3)	3 (2–4)	3 (3–5)
Ventral attention network	2 (1–3)	2 (1–3)	2 (1–3)	2 (1–3)
Visual network	1 (0–2)	1 (0–2)	1 (0–2)	1 (0–2)
Limbic network	1 (0–2)	1 (1–2)	1 (0–2)	1 (0–2)
Frontoparietal network	1 (0–1)	1 (0–2)	1 (1–2)	1 (0–1)

*Values are presented as median [interquartile range (IQR)] of all subjects, as well as of a young, middle-aged, and old group.*

## Discussion

To our knowledge, this is the first study to explore the effect of age on individual brain metabolic connectivity using [^18^F]FDG PET. Our results showed that individual brain metabolic networks became less integrated and less segregated during aging. We observed these age-related effects on the level of the whole brain as well as within the functional subnetworks except within the control, limbic, and visual network. The same decrease of metabolic connectivity was found between several networks across the whole-brain network and the functional subnetworks. Meanwhile, no network reorganization was observed with aging as distribution of hubs throughout the brain, and different subnetworks remained unchanged during aging.

In literature, only few studies explored the effect of aging on metabolic connectivity networks in healthy subjects. While our study found a decrease in metabolic connectivity with aging, two other groups reported an opposite trend using a group-based correlation approach (Arnemann et al., 2018; Huang et al., 2021). However, it is not straightforward to compare our findings with these studies as a group-based correlation approach highly depends on group composition where a homogeneous group with low inter-subject variability in regional [^18^F]FDG uptake could result in lower correlation measures. In addition, correlation measures are not sensitive to differences in regional uptake values which are consistent across subjects. On the contrary, the KLSE approach compares the intra-regional metabolic distribution between different regions within a single subject where it uses all uptake values within each brain region and thus much more information compared with correlation-based measures which consider only the averaged regional uptake of brain regions. As such, this approach provides a quantitative representation of the tracer distribution throughout the brain and the different subnetworks with a high average metabolic strength between nodes representing a rather homogeneous [^18^F]FDG uptake in the corresponding brain regions. This way, metabolic connectivity estimated across subjects using correlation measures and with-subject using the KLSE approach proved to be high complementary. Meanwhile, univariate VOI-based and voxelwise approaches have mainly been used so far to study age-related effects on brain glucose metabolism. These studies showed differences in the age-dependency of glucose metabolism between different brain regions with consistently higher age-related decrease of [^18^F]FDG uptake in the frontal, cingulate, temporal cortex, and in subcortical GM regions compared with other brain regions (Fujimoto et al., 2008; Knopman et al., 2014; Yoshizawa et al., 2014; Kakimoto et al., 2016; Ishibashi et al., 2017; Malpetti et al., 2017; Van Aalst et al., 2021). As this translates into a more heterogeneous distribution of [^18^F]FDG uptake throughout the brain with aging, this also results in a lower global metabolic connectivity strength in elderly healthy persons which is in line with our findings. More specifically, these VOI-based results, showing higher age-related decrease of [^18^F]FDG uptake in prefrontal cortex, medial frontal cortex, temporal and high parietal cortex, and insula (Van Aalst et al., 2021), also support our findings of an age-related decrease of metabolic connectivity in default mode, frontoparietal, control, dorsal attention, and ventral attention network as these brain regions are involved in these functional networks.

Contrary to metabolic connectivity, the effect of age on functional connectivity has been extensively explored using fMRI (Betzel et al., 2014; Geerligs et al., 2014; Damoiseaux, 2017; Farras-Permanyer et al., 2019; Varangis et al., 2019; Mancho-Fora et al., 2020). In general, functional connectivity based on fMRI showed an age-related decrease in functional connectivity and a loss in network integrity as well as in network segregation during the lifespan, which are in line with our metabolic connectivity findings. Within subnetworks, Varangis et al. (2019) reported decreased functional connectivity based on fMRI in the default mode, frontoparietal, ventral attention, and dorsal attention network which is similar as our age-related changes within the functional subnetworks using [^18^F]FDG PET. To compare our findings with these fMRI studies on functional connectivity, we used the Schaefer atlas for the brain parcellation as this allowed us to define the major functional networks across the cerebral cortex which are also frequently used in fMRI analysis (Watabe and Hatazawa, 2019). Although connectivity metrics are not directly comparable, we observed a similar decrease in local efficiency and increase in characteristic path length and clustering coefficient (Sun et al., 2012).

In terms of methodology, we applied a novel KLSE approach to define metabolic connectivity at a subject level. This approach has already been validated and successfully implemented to predict the progression from MCI to AD using [^18^F]FDG PET (Wang et al., 2020a,b). To determine an individual metabolic network, the KLSE approach relies on a predefined atlas for brain parcellation and defining the nodes of each individual network. To assure a robust estimation of the PDF within each parcel, a granularity of 100 parcels was selected for the whole-brain parcellation as more parcels would result in less voxels per parcel and impact PDF estimates (Brownlee, 2020). In addition, a fully weighted network approach was chosen to preserve the higher information content over binary network and avoid the need for a rather arbitrary threshold for the binarization. Although weighted networks are often more difficult to interpret, weighted networks are especially of interest for studying brain metabolic connectivity as variations in metabolic connectivity strength can be described by connectivity weights (Fornito et al., 2016). Finally, we also implemented the KLSE approach including a region-based voxelwise correction for partial volume effects (PVC) to assess the contribution of underlying morphology changes on aging (Thomas et al., 2011; Greve et al., 2016). Age-related results from metabolic connectivity based on PVC [^18^F]FDG PET images also showed a less integrated and less segregated metabolic brain network during aging within the whole brain and the same functional subnetworks (Supplementary Table 3). In 18 out of 36 (50%) between-networks, a similar age-related decrease in metabolic connectivity was found across the whole brain and several functional subnetworks (Supplementary Table 4). Altogether, age-related results from metabolic connectivity based on PVC [^18^F]FDG PET images agreed with the uncorrected PET FDG results (Supplementary Table 3), suggesting a true observed effect of aging on the metabolic connectivity.

In terms of study limitations, we considered only a limited number of connectivity metrics. However, we made sure to include metrics that represented both global and local brain metabolic connectivity and measured network integration, segregation, and centrality such that a wide range of connectivity metrics was covered. Another limitation is that we did not look at a gender effect on the metabolic connectivity due to rather small sample size and because man and women were not homogeneously distributed within our study population.

In the future, it would be interesting to further explore this novel individual approach for PET tracers targeting specific neurotransmission systems, amyloid load, or tau deposition (Sala and Perani, 2019). For these tracers, single-subject network metrics could serve as diagnostic markers to quantify differences between healthy subjects and specific patient groups and to explore the association between these individual metrics and clinical outcome (Paldino et al., 2017; Fortier et al., 2019). However, a diagnostic approach would probably benefit much more from using a brain atlas for the parcellation, which is more related to PET data analysis, such as the Hammers atlas, instead of the Schaefer atlas which is more functionally oriented. Furthermore, one could take advantage of this individual approach and combine this individual metabolic network with individual structural and functional networks to obtain an integrated multiplex network. This way, the network topology of the human brain can be explored from a multilayer perspective which could further improve diagnosis and patient stratification (Giuliano Zippo and Castiglioni, 2016).

## Conclusion

In this study, age-related changes in brain metabolic connectivity during the adult lifespan were revealed using an individual brain metabolic network constructed with [^18^F]FDG PET. Overall, metabolic connectivity within the whole-brain network decreased with aging and resulted into a less integrated and less segregated network, while no evidence was found for reorganization of brain metabolic networks during healthy aging. The same age-related decrease in metabolic connectivity was also found in predefined functional subnetworks but not in the control, limbic, and visual network. A similar decrease in metabolic connectivity with aging was observed between several networks across the whole brain and functional subnetworks. Finally, these findings were in line with age-related functional connectivity changes during the adult lifespan using fMRI.

## Data Availability Statement

The data that support the findings of this study are not publicly available due to privacy/ethical restrictions. Upon reasonable request, the anonymized data could be shared on approval by the local ethics committee. Requests to access the datasets should be directed to NM, nathalie.mertens@kuleuven.be.

## Ethics Statement

The studies involving human participants were reviewed and approved by the Local Ethics Committee of UZ Leuven Gasthuisberg. The patients/participants provided their written informed consent to participate in this study.

## Author Contributions

NM and MK contributed to the analysis of data and design of this study. NM, SS, KVL, and MK contributed to interpretation of data and design of this study. All authors critically revised the manuscript.

## Conflict of Interest

NM is Ph.D. mandate holder of the Fund for Scientific Research, Flanders, Belgium (FWO, 1S57420N). KVL has conducted this study as senior clinical research fellow for the Research Foundation Flanders (FWO). The remaining authors declare that the research was conducted in the absence of any commercial or financial relationships that could be construed as a potential conflict of interest.

## Publisher’s Note

All claims expressed in this article are solely those of the authors and do not necessarily represent those of their affiliated organizations, or those of the publisher, the editors and the reviewers. Any product that may be evaluated in this article, or claim that may be made by its manufacturer, is not guaranteed or endorsed by the publisher.
