# Supplementary material for: The Effect of Aging on Brain Glucose Metabolic Connectivity Revealed by [18F]FDG PET-MR and Individual Brain Networks
Source: Front Aging Neurosci. 2022 Feb 9;13:798410. doi: 10.3389/fnagi.2021.798410 (PMC8865456; doi:10.3389/fnagi.2021.798410)
Supplement: Supplementary file 1 [file Data_Sheet_1.docx]

Supplementary Material

# Supplementary Figures

Supplementary Figure 1: Mean and coefficient of variation of the individual metabolic connectivity strength for a young, middle-aged and old subject group.

*
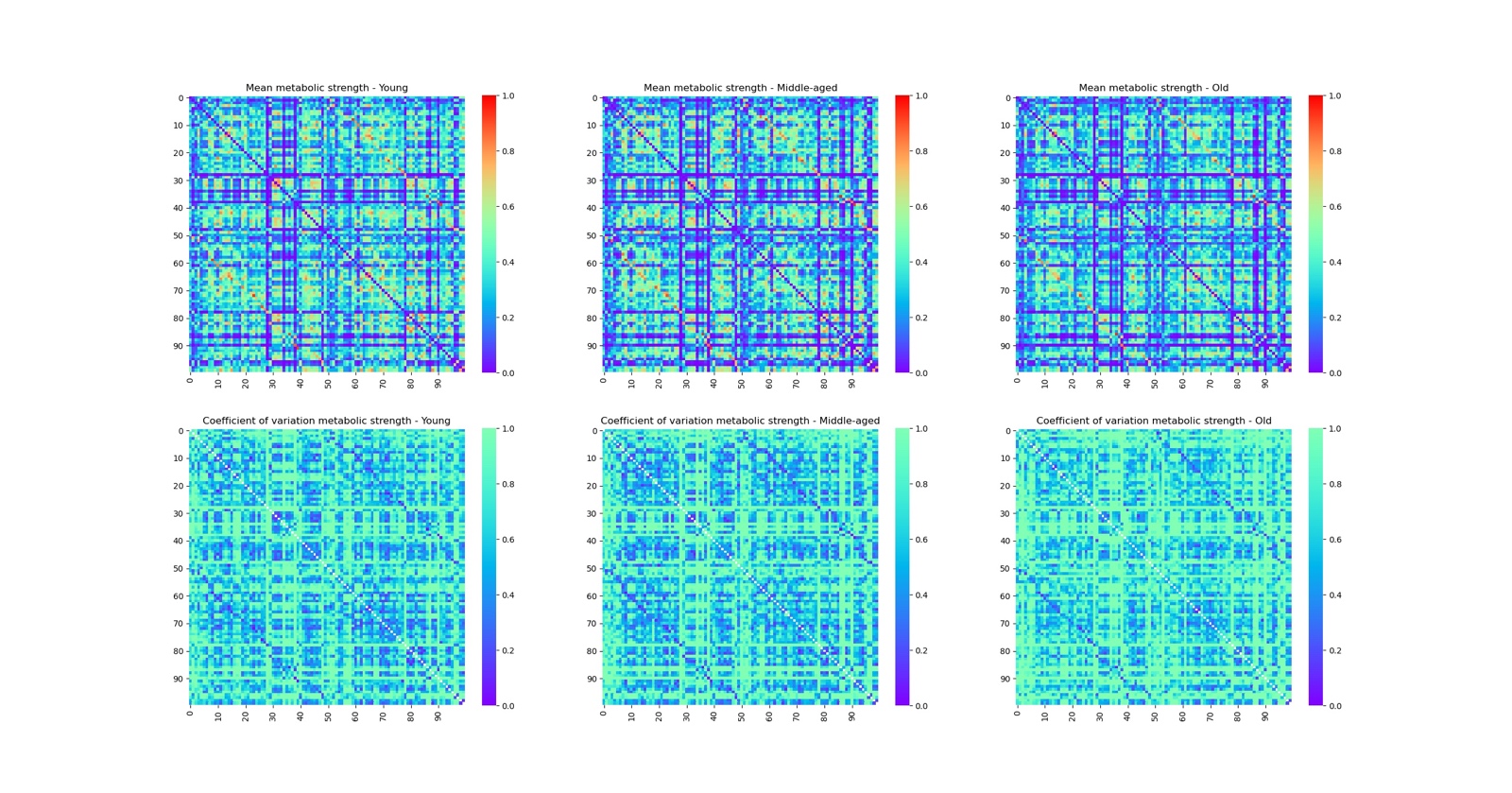
*

Supplementary Figure 2: Normalized distribution of metabolic strength of a 20-year-old and an 80-year-old subject, showing a shift towards lower values for the 80-year-old subject compared to the 20-year-old subject.


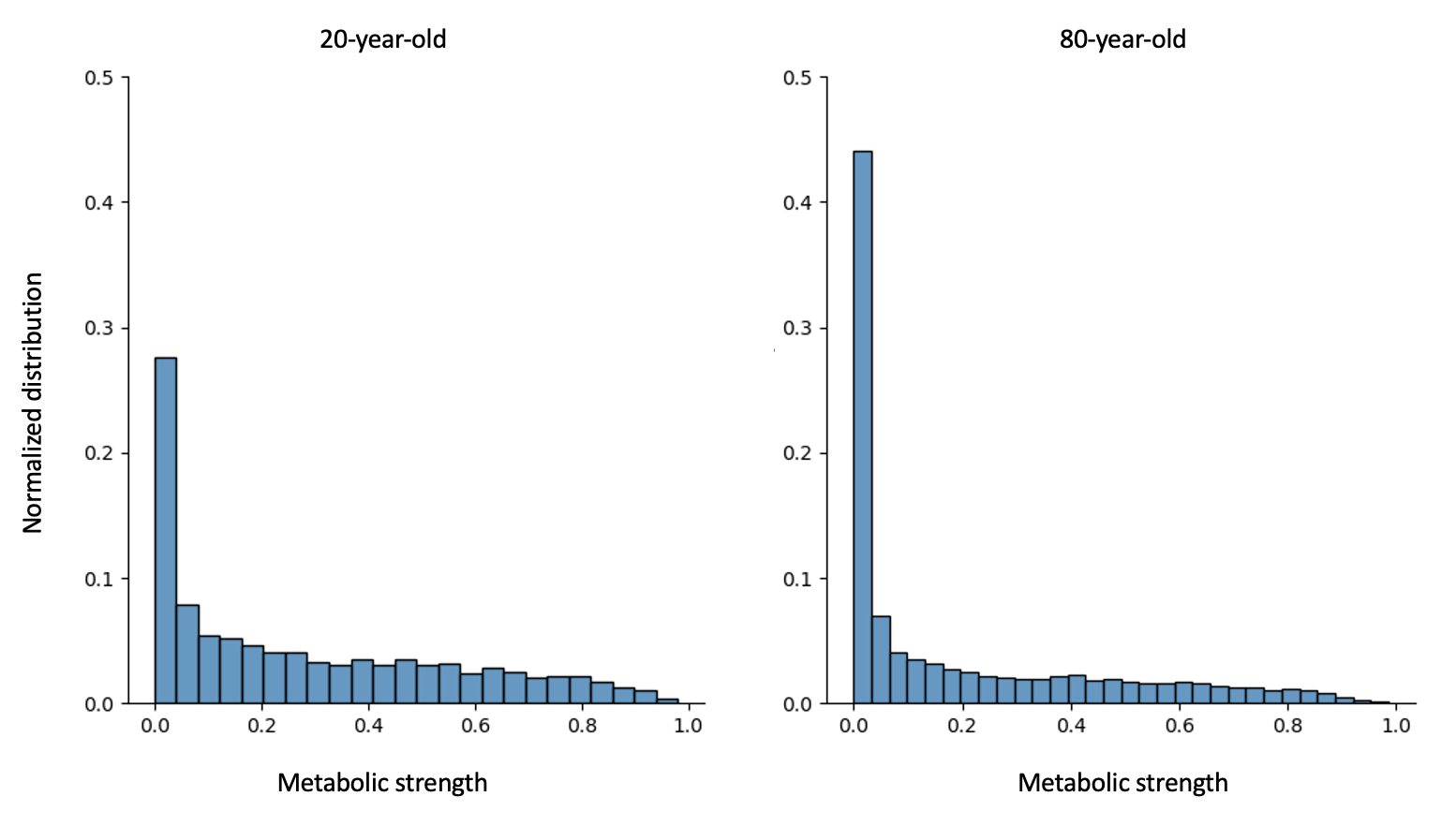


# Supplementary Tables

Supplementary Table 1: Overview of nodes for each functional subnetwork obtained based on parcels of the Schaefer atlas, consisting of the frontoparietal network and 7 functional networks.

| Functional network | Indices of the corresponding brain parcels (left; right hemisphere) |
| --- | --- |
| Visual network | 0-6; 50-55 |
| Somatomotor network | 7-12; 56-63 |
| Dorsal attention network | 13-19; 64-69 |
| Ventral attention network | 20-26; 70-76 |
| Limbic network | 27-29; 77-78 |
| Control network | 30-36; 79-87 |
| Default mode network | 37-48; 88-96 |
| Frontoparietal network | 49; 97-99 |

Supplementary Table 2: Overview of multiple linear regression models between metabolic connectivity strength with age between networks. Multiple linear regressions are described as Y = ß0 + ß1.age + ß2.age^2^. Regression p-values, overall r-values and regression coefficients are given. Significant p-values and coefficients are given in bold. Metric values and differences between an 80-year-old and 20-year-old male are also given.

| **Metabolic connectivity strength** | p-value | r-value | ß0 | ß1 | ß2 | 20y | 80y | %Diff |
| --- | --- | --- | --- | --- | --- | --- | --- | --- |
| Visual – frontoparietal | **0.0006** | 0.46 | **0.20** | 3.85E-03 | **-0.05E-03** | 0.26 | 0.17 | -33.2 |
| Somatomotor – dorsal attention | **0.0028** | 0.41 | **0.36** | 3.87E-03 | **-0.05E-03** | 0.42 | 0.34 | -17.8 |
| Somatomotor – frontoparietal | **0.0019** | 0.42 | **0.25** | **6.04E-03** | **-0.07E-03** | 0.34 | 0.28 | -17.7 |
| Dorsal attention – frontoparietal | **<0.0001** | 0.58 | **0.31** | **6.03E-03** | **-0.08E-03** | 0.40 | 0.29 | -28.0 |
| Ventral attention – frontoparietal | **0.0011** | 0.44 | **0.26** | 5.67E-03 | **-0.07E-03** | 0.35 | 0.27 | -23.9 |
| Default mode – frontoparietal | **0.0013** | 0.43 | **0.21** | **5.89E-03** | **-0.07E-03** | 0.30 | 0.24 | -18.5 |
| Frontoparietal – whole brain | **<0.0001** | 0.54 | **0.24** | **4.93E-03** | **-0.06E-03** | 0.31 | 0.24 | -22.9 |
| Visual – whole brain | **0.0098** | 0.31 | **0.28** | **-0.74E-03** | / | 0.27 | 0.23 | -16.5 |
| Visual – default mode | **0.0255** | 0.27 | **0.26** | **-0.65E-03** | / | 0.25 | 0.21 | -15.5 |
| Visual – dorsal attention | **0.0086** | 0.32 | **0.35** | **-1.10E-03** | / | 0.32 | 0.26 | -20.3 |
| Visual – limbic | **0.0001** | 0.45 | **0.25** | **-1.85E-03** | / | 0.21 | 0.10 | -53.0 |
| Visual – ventral | **0.0131** | 0.30 | **0.30** | **-0.99E-03** | / | 0.28 | 0.22 | -21.3 |
| Somatomotor – whole brain | **0.0338** | 0.26 | **0.35** | **-0.63E-03** | / | 0.33 | 0.30 | -11.3 |
| Dorsal attention – whole brain | **0.0010** | 0.39 | **0.38** | **-1.07E-03** | / | 0.36 | 0.29 | -18.0 |
| Dorsal attention – limbic | **0.0034** | 0.35 | **0.32** | **-1.56E-03** | / | 0.29 | 0.20 | -32.0 |
| Dorsal attention – ventral attention | **0.0004** | 0.42 | **0.45** | **-1.74E-03** | / | 0.41 | 0.31 | -25.2 |
| Ventral attention – whole brain | **<0.0001** | 0.48 | **0.38** | **-1.17E-03** | / | 0.36 | 0.29 | -19.8 |
| Ventral attention – control | **0.0036** | 0.35 | **0.37** | **-1.16E-03** | / | 0.35 | 0.28 | -19.9 |
| Ventral attention – default mode | **0.0006** | 0.41 | **0.40** | **-1.20E-03** | / | 0.37 | 0.30 | -19.3 |
| Ventral attention – limbic | **0.0034** | 0.35 | **0.32** | **-1.56E-03** | / | 0.29 | 0.20 | -32.0 |
| Limbic – whole brain | **0.0013** | 0.38 | **0.25** | **-1.20E-03** | / | 0.22 | 0.15 | -32.0 |
| Limbic – default mode | **0.0463** | 0.24 | **0.23** | **-0.69E-03** | / | 0.22 | 0.17 | -19.2 |
| Limbic – frontoparietal | **<0.0001** | 0.55 | **0.35** | **-2.12E-03** | / | 0.30 | 0.18 | -41.8 |
| Control – whole brain | **0.0297** | 0.27 | **0.30** | **-0.65E-03** | / | 0.29 | 0.25 | -13.4 |
| Control – default mode | **0.0002** | 0.45 | **0.36** | **-1.14E-03** | / | 0.34 | 0.27 | -20.2 |
| Default mode – whole brain | **0.0018** | 0.38 | **0.34** | **-0.83E-03** | / | 0.32 | 0.27 | -15.6 |
| Visual – control | 0.8348 | / | / | / | / | / | / | / |
| Visual – somatomotor | 0.2547 | / | / | / | / | / | / | / |
| Somatomotor – control | 0.7605 | / | / | / | / | / | / | / |
| Somatomotor – default mode | 0.2320 | / | / | / | / | / | / | / |
| Somatomotor – limbic | 0.0663 | / | / | / | / | / | / | / |
| Somatomotor – ventral | 0.1730 | / | / | / | / | / | / | / |
| Dorsal attention – control | 0.2546 | / | / | / | / | / | / | / |
| Dorsal attention – default mode | 0.1550 | / | / | / | / | / | / | / |
| Limbic – control | 0.2318 | / | / | / | / | / | / | / |
| Control – frontoparietal | 0.4449 | / | / | / | / | / | / | / |

Supplementary Table 3: Overview of multiple linear regression analyses after partial volume correction (PVC) to model network metrics as function of age within the whole brain network and within functional subnetworks. Multiple linear regressions are described as Y = ß0 + ß1.age + ß2.age2. Regression p-values, overall r-values and regression coefficients are given. Significant p-values and coefficients are given in bold. Metric values and differences between an 80-year-old and 20-year-old subject are also given.

|  | p-value | | r-value | ß0 | ß1 | ß2 | 20y | 80y | %Diff |
| --- | --- | --- | --- | --- | --- | --- | --- | --- | --- |
| **Mean connectivity strength** | |  |  |  |  |  |  |  |  |
| Whole brain network | **0.0086** | | 0.33 | **0.39** | **-0.69E-03** | / | 0.37 | 0.33 | -11.1 |
| Frontoparietal network | **0.0008** | | 0.46 | **0.21** | 5.39E-03 | **-0.07E-03** | 0.29 | 0.21 | -28.4 |
| Default mode network | **0.0205** | | 0.29 | **0.38** | **-0.85E-03** | / | 0.36 | 0.31 | -14.2 |
| Control network | **<0.0001** | | 0.47 | **0.53** | **-1.49E-03** | / | 0.50 | 0.41 | -17.8 |
| Dorsal attention network | **0.0134** | | 0.31 | **0.58** | **-1.38E-03** | / | 0.56 | 0.47 | -14.9 |
| Ventral attention network | **<0.0001** | | 0.50 | **0.50** | **-2.12E-03** | / | 0.46 | 0.33 | -27.5 |
| Somatomotor network | 0.6641 | | / | / | / | / | / | / | / |
| Limbic network | 0.5845 | | / | / | / | / | / | / | / |
| Visual network | 0.1273 | | / | / | / | / | / | / | / |
| **Characteristic path length** | |  |  |  |  |  |  |  |  |
| Whole brain network | **0.0022** | | 0.38 | **2.10** | **3.26E-03** | / | 2.16 | 2.36 | 9.1 |
| Frontoparietal network | **<0.0001** | | 0.53 | **3.21** | **-49.28E-03** | **0.62E-03** | 2.47 | 3.24 | 31.3 |
| Dorsal attention network | **0.0033** | | 0.41 | **1.90** | -13.67E-03 | **0.18E-03** | 1.70 | 1.93 | 13.8 |
| Default mode network | **0.0065** | | 0.34 | **2.14** | **5.16E-03** | / | 2.25 | 2.56 | 13.8 |
| Control network | **0.0002** | | 0.46 | **1.67** | **4.55E-03** | / | 1.76 | 2.03 | 15.5 |
| Ventral attention network | **<0.0001** | | 0.52 | **1.67** | **9.63E-03** | / | 1.86 | 2.44 | 31.0 |
| Somatomotor network | 0.6906 | | / | / | / | / | / | / | / |
| Limbic network | 0.3810 | | / | / | / | / | / | / | / |
| Visual network | 0.1648 | | / | / | / | / | / | / | / |
| **Average clustering coefficient** | |  |  |  |  |  |  |  |  |
| Whole brain network | **0.0086** | | 0.33 | **0.41** | **-0.73E-03** | / | 0.39 | 0.35 | -11.2 |
| Frontoparietal network | **0.0102** | | 0.32 | **0.54** | **-1.68E-03** | / | 0.51 | 0.41 | -19.7 |
| Default mode network | **0.0176** | | 0.30 | **0.42** | **-0.94E-03** | / | 0.40 | 0.34 | -14.2 |
| Control network | **<0.0001** | | 0.47 | **0.60** | **-1.64E-03** | / | 0.56 | 0.47 | -17.4 |
| Dorsal attention network | **0.0194** | | 0.29 | **0.67** | **-1.48E-03** | / | 0.64 | 0.55 | -14.0 |
| Ventral attention network | **<0.0001** | | 0.48 | **0.58** | **-2.27E-03** | / | 0.53 | 0.40 | -25.6 |
| Somatomotor network | 0.6189 | | / | / | / | / | / | / | / |
| Limbic network | 0.6429 | | / | / | / | / | / | / | / |
| Visual network | 0.0610 | | / | / | / | / | / | / | / |
| **Average local efficiency** | |  |  |  |  |  |  |  |  |
| Whole brain network | **0.0133** | | 0.31 | **0.28** | **-0.40E-03** | / | 0.27 | 0.24 | -8.9 |
| Frontoparietal network | **0.0016** | | 0.43 | 0.09 | **9.05E-03** | **-0.10E-03** | 0.23 | 0.14 | -37.1 |
| Default mode network | **0.0577** | | 0.30 | **0.17** | 2.93E-03 | **-0.03E-03** | 0.21 | 0.20 | -7.7 |
| Control network | **0.0050** | | 0.34 | **0.35** | **-0.87E-03** | / | 0.33 | 0.28 | -15.6 |
| Dorsal attention network | **0.0454** | | 0.25 | **0.42** | **-0.85E-03** | / | 0.40 | 0.35 | -12.7 |
| Ventral attention network | **0.0002** | | 0.45 | **0.31** | **-1.38E-03** | / | 0.29 | 0.20 | -29.0 |
| Somatomotor network | 0.4942 | | / | / | / | / | / | / | / |
| Limbic network | 0.3843 | | / | / | / | / | / | / | / |
| Visual network | 0.1552 | | / | / | / | / | / | / | / |

Supplementary Table 4: Overview of multiple linear regression models after partial volume correction (PVC) to model between metabolic connectivity strength with age between networks. Multiple linear regressions are described as Y = ß0 + ß1.age + ß2.age^2^. Regression p-values, overall r-values and regression coefficients are given. Significant p-values and coefficients are given in bold. Metric values and differences between an 80-year-old and 20-year-old male are also given.

| **Metabolic connectivity strength** | p-value | r-value | ß0 | ß1 | ß2 | 20y | 80y | %Diff |
| --- | --- | --- | --- | --- | --- | --- | --- | --- |
| Visual – frontoparietal | **0.0225** | 0.34 | 0.28 | **5.38E-03** | **-0.06E-03** | 0.36 | 0.31 | -14.5 |
| Dorsal attention – whole brain | **0.0098** | 0.37 | 0.34 | 3.56E-03 | **-0.04E-03** | 0.39 | 0.35 | -11.2 |
| Dorsal attention – frontoparietal | **<0.0001** | 0.54 | 0.31 | **8.86E-03** | **-0.10E-03** | 0.44 | 0.36 | -17.7 |
| Ventral attention – frontoparietal | **0.0012** | 0.44 | 0.36 | 4.50E-03 | **-0.06E-03** | 0.43 | 0.34 | -20.2 |
| Default mode – frontoparietal | **0.0027** | 0.42 | 0.23 | **7.63E-03** | **-0.08E-03** | 0.35 | 0.31 | -12.2 |
| Frontoparietal – whole brain | **0.0003** | 0.48 | 0.28 | **5.81E-03** | **-0.07E-03** | 0.37 | 0.31 | -14.9 |
| Visual – limbic | **0.0004** | 0.43 | 0.37 | **-1.98E-03** | / | 0.33 | 0.21 | -36.3 |
| Somatomotor – limbic | **0.0406** | 0.25 | 0.37 | **-1.30E-03** | / | 0.34 | 0.26 | -22.9 |
| Dorsal attention – limbic | **0.0494** | 0.24 | 0.41 | **-1.17E-03** | / | 0.39 | 0.32 | -17.9 |
| Dorsal attention – ventral attention | **0.0017** | 0.38 | 0.51 | **-1.60E-03** | / | 0.47 | 0.38 | -20.3 |
| Ventral attention – whole brain | **0.0008** | 0.41 | 0.42 | **-1.04E-03** | / | 0.40 | 0.34 | -15.7 |
| Ventral attention – control | **0.0021** | 0.37 | 0.40 | **-1.35E-03** | / | 0.37 | 0.29 | -21.6 |
| Ventral attention – default mode | **0.0009** | 0.40 | 0.44 | **-1.22E-03** | / | 0.42 | 0.34 | -17.5 |
| Ventral attention – limbic | **0.0494** | 0.24 | 0.41 | **-1.17E-03** | / | 0.39 | 0.32 | -17.9 |
| Limbic – whole brain | **0.0058** | 0.34 | 0.32 | **-1.11E-03** | / | 0.30 | 0.23 | -22.1 |
| Limbic – frontoparietal | **0.0056** | 0.34 | 0.41 | **-1.43E-03** | / | 0.38 | 0.29 | -22.7 |
| Control – default mode | **0.0016** | 0.38 | 0.41 | **-1.09E-03** | / | 0.39 | 0.32 | -16.8 |
| Visual – whole brain | 0.9078 | / | / | / | / | / | / | / |
| Visual – control | 0.0634 | / | / | / | / | / | / | / |
| Visual – default mode | 0.5622 | / | / | / | / | / | / | / |
| Visual – dorsal attention | 0.0595 | / | / | / | / | / | / | / |
| Visual – somatomotor | 0.9915 | / | / | / | / | / | / | / |
| Visual – ventral | 0.5422 | / | / | / | / | / | / | / |
| Somatomotor – whole brain | 0.3770 | / | / | / | / | / | / | / |
| Somatomotor – control | 0.1216 | / | / | / | / | / | / | / |
| Somatomotor – default mode | 0.9284 | / | / | / | / | / | / | / |
| Somatomotor – dorsal attention | 0.0844 | / | / | / | / | / | / | / |
| Somatomotor – frontoparietal | 0.4454 | / | / | / | / | / | / | / |
| Somatomotor – ventral | 0.4818 | / | / | / | / | / | / | / |
| Dorsal attention – control | 0.5245 | / | / | / | / | / | / | / |
| Dorsal attention – default mode | 0.5287 | / | / | / | / | / | / | / |
| Limbic – control | 0.1005 | / | / | / | / | / | / | / |
| Limbic – default mode | 0.1913 | / | / | / | / | / | / | / |
| Control – whole brain | 0.1813 | / | / | / | / | / | / | / |
| Control – frontoparietal | 0.2014 | / | / | / | / | / | / | / |
| Default mode – whole brain | 0.0937 | / | / | / | / | / | / | / |
